# Goloba kusi (*Hornstedtia scottiana* [F. Muell.] K. Schum.) fruit as a feed additive to improve the histological structures and growth performance of broiler

**DOI:** 10.14202/vetworld.2023.329-340

**Published:** 2023-02-17

**Authors:** D. Blatama, N. Salsabila, H. T. Saragih

**Affiliations:** 1 Post Graduate Program of Biology, Department of Tropical Biology, Universitas Gadjah Mada, Yogyakarta, Indonesia; 2 Graduate Program of Biology, Department of Tropical Biology, Universitas Gadjah Mada, Yogyakarta, Indonesia; 3 Laboratory of Animal Development Structure, Faculty of Biology, Universitas Gadjah Mada, Yogyakarta, Indonesia

**Keywords:** feed additive, Goloba kusi fruit, growth performance, muscle morphology, small intestine

## Abstract

**Background and Aim:**

The broiler farming industry in Indonesia has enormous potential, with chicken meat that can be optimized by providing adequate and high-quality feed. However, the main raw material for the feed still relies on imported products, which makes it necessary to produce alternative materials from native plants. Therefore, this study aimed to investigate the effect of giving Goloba kusi fruit (GF) (*Hornstedtia scottiana* [F. Muell.] K. Schum.) on the growth of the small intestine, pectoralis major, and gastrocnemius muscle, as well as the development of broiler chickens.

**Materials and Methods:**

This study used a completely randomized design, in which 300 day-old Chicks were divided into five groups, consisting of 12 chickens in each group with five replications. The GF treatments, namely, 0% (control [CON]), 0.625% (GF1), 1.25% (GF2), 2.5% (GF3), and 5% (GF4) were administered through per kg basal feed. Subsequently, three chickens from each replication were taken, decapitated on the neck, subjected to surgery for histological preparations, and stained with Hematoxylin-Eosin and Periodic acid-Schiff-alcian blue. The variables observed included small intestine morphology, muscle morphology, and chicken growth performance.

**Results:**

The results showed that the small intestine morphology, muscle morphology, and chicken growth performance of the GF4 (5%) group increased significantly compared to the CON group.

**Conclusion:**

The administration of GF with an optimum concentration of 5% through basal feed improves small intestine morphology, muscle morphology, and chicken growth performance.

## Introduction

There is an urgent need for continuous research into the available unconventional feed additions to save the poultry industry, particularly in developing countries [1]. The use of native flora, such as spices of plants from the *Zingiberaceae* family has been practiced for a long time as one of the ingredients for medicine, feed, drinks, and cooking spices by Indonesians [2]. *Zingiberaceae* has members from about 47 genera and 1400 species [3] with high antioxidant content and can function as an anticancer [4]. The eastern region of the country has a distinctive plant not owned by the western region, namely, *Hornstedtia scottiana* (F. Muell.) K. Schum. or Goloba kusi fruit (GF) (vernacular name) [5, 6]. The GF is mostly found in Sulawesi, Maluku, and Papua and is often used in traditional medicine, cooking ingredients, and antimalarial medicine [7]. Furthermore, it belongs to the *Zingiberaceae* family, is rhizome-shaped, and can thrive in the wet tropics, including in forest areas [5]. It can also grow up to 4 m in height [8]; the fruit has numerous seeds, and individual capsules that are 10–25 mm long. The key features are capsules held within the red and white cone-like structure, which occur close to the ground and arise separately from the leaves [5, 6].

Plants belonging to *Zingiberaceae* have been widely used as potential functional feeds [9] and raw materials for livestock feed. One of this livestock is chicken meat from broilers, that is widely consumed by Indonesians. This is due to the more economical price compared to other livestock meat, the short production time, and the quality of the meat with good protein content. However, producing optimal chicken meat quality requires relatively expensive feed costs [10], which is the main obstacle often faced by the farming industry. Until now, chicken feed on the market is still heavily on imported raw materials such as soybean meal and corn [11]. This makes it necessary to find alternative raw materials to manufacture chicken feed and avoid dependence on imported materials. This can be achieved by using vegetable protein derived from plants in Indonesia. Protein with all its amino acids is very important for broilers and layers to achieve growth performance and maintain optimal health, especially during diseases or heat stress [12]. It was also discovered that secondary metabolites, namely, flavonoids from plants, can improve the quality of poultry products [13].

Based on this explanation, GF can be used as an alternative raw material for broiler chicken feed but there are no reports on its application. Therefore, this study was conducted to determine the effect of GF as a feed additive on the growth of the small intestine, pectoralis major muscle, gastrocnemius muscle, and growth performance of broiler chickens.

## Materials and Methods

### Ethical approval

This study obtained a code of ethics from the Faculty of Veterinary Medicine, Gadjah Mada University, and has been declared to meet the ethical requirements for conducting research with certification number 00141/EC-FKH/Eks/2021.

### Study period and location

The study was conducted from March to June 2022 at Sawitsari Research Station and Laboratory of Animal Development Structure, Faculty of Biology, Universitas Gadjah Mada, Indonesia.

### Proximate analysis, calorie, and flavonoid test

The proximate and calorie tests were carried out in a certified laboratory, namely, the Center for Feed and Nutrition Studies, Gadjah Mada University. The proximate test was conducted to determine the composition and nutritional content of the GF, while the calorie test was used to examine the calorie contents [14, 15]. The flavonoid test was performed according to Chandra *et al*. [16] in a certified laboratory, namely, LPPT Gadjah Mada University.

### Feed preparation

The GF was sourced from Halmahera Island, North Maluku, Indonesia, and identified by the Head of the Plant Systematics Laboratory, Faculty of Biology, UGM, Prof. Dr. Purnomo. Goloba kusi fruit was dried at room temperature (30°C) and mashed using a blender to obtain a smooth sample, which was mixed with basal feed obtained from industrial partners, as presented in Table-1 [17]. A total of 300 male and female day-old chicks (DOC) were used to investigate the effect of GF addition in the basal feed. The study was conducted for 21 days, with an average body weight of 46–47 g. Day-old chicks were randomly divided into five treatment groups with five replicates per group, consisting of 12 chickens in each replicate of the group, under 24-h light conditions. The groups included the control (CON) group without addition and treatment groups, namely, GF1, GF2, GF3, and GF4 with the addition of 0.625%/kg, 1.25%/kg, 2.5%/kg, and 5%/kg basal feed, respectively. The mixture of GF and basal feed was made for four treatment groups based on the percentage of the fruit mixed in the basal feed.

**Table-1:** Basal feed formulation and nutrition content.

Composition of feed (%)	Single feed
Corn	49.0
Soybean meal	29.0
Rice bran	9.8
Full-fat soya	5.4
Crude palm oil	3.0
Dicalcium phosphate	2.37
Premix vitamin^a^	0.03
Premix mineral^b^	0.06
D, L-methionine	0.22
NaCl	0.32
Calcit	0.5
L-lysine HCl	0.1
L-threonine	0.04
Choline chloride 60%	0.16
Calculated composition^c^	
Metabolizable energy of poultry (kcal/kg)	2,904.02
Crude protein (%)	20.23
Crude fat (%)	8.30
Fiber (%)	3.37
Lysine (%)	1.22
Methionine (%)	0.53
Methionine + cysteine (%)	0.86
Calcium (%)	1
Phosphorus, total (%)	0.95
Phosphorus, available (%)	0.5
Sodium (%)	0.15
Chloride (%)	0.23

a Premix vitamin provided the following per kilogram of diet (Vitamin A: 15000 IU, Vitamin D_3_: 3000 IU, Vitamin E: 22.5 mg, Vitamin K_3_: 3 mg, Vitamin B_1_: 3 mg, Vitamin B_2_: 9 mg, Vitamin B_6_: 4.5 mg, Vitamin B_12_: 30 mcg, biotin: 30 mcg, folic acid: 1.5 mg, niacin: 45 mg, pantothenic acid: 1.5 mg, Vitamin C: 0 mg, choline: 2090 mg and 1242 mg),

b Premix mineral provided the following per kilogram of diet (Cu: 12 mg, Fe: 72 mg, Iodine: 0.9 mg, Mn: 84 mg, Se: 0.3 mg, Zn: 60 mg),

c Proximate, amino acids, minerals, and Metabolizable energy were obtained from calculated values [17]

### Acclimation, feeding, and maintenance

Acclimation was carried out for 3 days from post-hatch to 3 days of age. Chickens were fed with basal feed, as shown in Table-1, and drank palm sugar water on the day of arrival. Subsequently, intensive maintenance was carried out in a container equipped with a light bulb, water, and feed supplemented with GF until 21 days. This was performed because, at age 21 days, the chickens are in the grower phase; therefore, it is good for growth performance study [18]. Chickens were fed and watered *ad libitum;* body weight was measured at intervals of 3 days, starting from 0 to 21 days of age, while body morphometry was measured at 1, 14, and 21 days of age.

### Chicken body morphometric measurement

Chicken body morphometry was measured at 0 and 21 days using a midline and caliper. The morphometric variables consisted of back length, chest circumference, as well as thigh length and circumference. Inside, the chest is measured using a caliper, and other variables are measured using the midline. The measurement of the morphometric characteristics of chickens was carried out to determine the benefits of adding GF to chicken growth. Morphometric character is one indicator that can be used to measure the growth performance of chickens. According to Lawrence and Fowler [19] and Lonergan *et al*. [20], the measurement that is often used to determine growth in domestic animals is live body weight and simple linear, which includes length, width, height, and circumference of body features.

### Measuring feed conversion ratio (FCR)

The amount of feed given was calculated every day until 21 days. Furthermore, the FCR was measured by calculating the amount of feed per kilogram needed (Feed Intake) to increase body weight per kilogram (Weight Gain). The FCR value, according to Panase and Mengumphan [21], can be obtained by the formula:


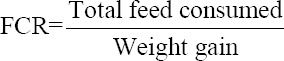


### Euthanasia, measurement of viscera organ index, muscle, and small intestine length

A total of three chickens aged 21 days from each replication were euthanized and the viscera of the heart, liver, spleen, pectoralis muscle tissue, gastrocnemius, and small intestine were taken. Measuring the index of viscera organs can be an indicator to know the balance of growth performance [22]. The left muscle tissue was collected to measure the muscle area, while the right muscle tissue was made for histological preparations. Meanwhile, the length of the duodenum and ileum was measured using the midline.

### Histological preparations of the pectoralis major, gastrocnemius, duodenum, and ileum muscles

Histological preparations of pectoralis major, gastrocnemius, duodenum, and ileum muscles that have been collected were made using the paraffin method. The right pectoralis major muscle was cut to a size of 1 × 1 cm and stained with hematoxylin-eosin (HE) to determine the area of the fasciculus and myofibers. The HE stain was used as basic histological staining that can differentiate nuclei from its surrounding [23]. Furthermore, observations were made under a Leica microscope with a magnification of 10× and 40× to observe fasciculus and myofibers, respectively. In the preparation, five fasciculus fields of view were taken and five myofibers were observed in each fasciculus.

To prepare the histology of the duodenum and ileum, the organs were cut transversely to observe the length of the villi and the depth of the crypts, and stained with Periodic acid Schiff-alcian blue (PAS-AB). The PAS-AB staining was used to detect neutral mucus in the goblet cells (PAS) and acid mucus (AB) [24–26]. Furthermore, villi, crypts, as well as goblet cells were observed and documented using a Leica microscope with 4× and 10× magnifications

Documentation results in myofiber area, fasciculus area, villi length, crypt depth, number, and goblet cells were measured using ImageJ software (National Institutes of Health and the Laboratory for Optical and Computational Instrumentation, LOCI, University of Wisconsin, USA).

### Statistical analysis

Statistical analysis was performed using a one-way analysis of variance, continued with Duncan’s test, and results were considered significant at p ≤ 0.05. All collected data were analyzed using the Statistical Package for the Social Sciences (SPSS 25.0) computer program (IBM SPSS, Inc., NY, USA) for windows.

## Results

### Small intestine morphology

**Figure-1:**
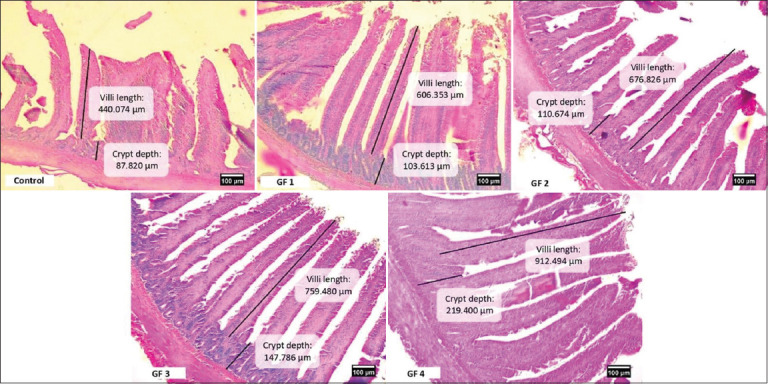
Transversal section villi and crypt of small intestine morphology of 21 days old broiler chicken treated with Goloba kusi fruit (GF) using 10 × 10 magnification. Control (CON): basal feed; GF1: GF1 0.625%/kg basal feed; GF2: GF2 1.25%/kg basal feed; GF3: GF3 2.5%/kg basal feed; GF4: GF4 5%/kg basal feed. This figure shows that the GF group had large size of villi and crypt compared to CON groups.

The morphology of the small intestine of broiler chickens treated with GF supplements is shown in Table-2 and Figure-1. The length of the duodenal villi in the GF3 and GF4 groups was significantly higher (p ≤ 0.05) compared to the CON group. Furthermore, the duodenal crypt depth also showed a significantly higher number (p ≤ 0.05). The duodenal villi/crypt ratio in the GF4 group was significantly (p ≤ 0.05) higher and produced the highest measure with the most significant value compared to the CON (p ≤ 0.05).

**Table-2:** Small intestine histology of broiler chickens fed with GF (*Hornstedtia scottiana*) treatments for 21 days.

Variables	Treatments					p-value

	Control	GF1	GF2	GF3	GF4	
Duodenum						
Villi length (μm)	893.936 ± 51.129^a^	999.794 ± 58.585^a^	1056.439 ± 42.635^a^	1343.471 ± 51.534^b^	1597.606 ± 66.015^c^	0.000
Crypt depth (μm)	127.890 ± 8.672^a^	125.923 ± 14.684^a^	148.434 ± 6.704^ab^	166.283 ± 5.554^b^	201.933 ± 13.303^c^	0.000
Villi/Crypt ratio	7.397 ± 0.824^a^	8.739 ± 1.202^a^	7.284 ± 0.517^a^	8.169 ± 0.453^a^	8.204 ± 0.613^a^	0.648
Number of goblet cells	157.667 ± 24.049^a^	198.111 ± 12.520^ab^	244.444 ± 9.427^b^	374.667 ± 26.004^c^	445.778 ± 36.634^d^	0.000
Goblet cells area (μm^2^)	27.067 ± 1.511^a^	31.301 ± 1.989^a^	59.345 ± 2.641^b^	77.741 ± 3.876^c^	86.613 ± 4.459^d^	0.000
Proximal ileum						
Villi length (μm)	528.204 ± 34.001^a^	692.115 ± 20.019^b^	761.609 ± 25.045^bc^	766.212 ± 29.056^bc^	836.346 ± 25.992^c^	0.000
Crypt depth (μm)	78.819 ± 5.488^a^	102.131 ± 3.966^b^	122.995 ± 7.029^bc^	124.395 ± 6.366^bc^	143.545 ± 12.171^d^	0.000
Villi/Crypt ratio	6.780 ± 0.342^a^	6.817 ± 0.207^a^	6.341 ± 0.370^a^	6.271 ± 0.372^a^	6.082 ± 0.405^a^	0.484
Number of goblet cells	148.222 ± 9.530^a^	182.444 ± 7.482^ab^	198.222 ± 20.495^b^	203.333 ± 10.493^b^	309.556 ± 14.298^c^	0.000
Goblet cells area (μm^2^)	26.720 ± 1.377^a^	40.884 ± 1.214^b^	46.057 ± 2.038^b^	64.947 ± 2.755^c^	73.222 ± 4.423^d^	0.000

Control: Basal feed, GF1: 0.625%/kg basal feed, GF2: 0.625%/kg basal feed, GF3: 0.625%/kg basal feed, GF4: 0.625%/kg basal feed. ^a-e^Different letters on values in the same row indicate a significant difference (p ≤ 0.05). *GF=Goloba kusi fruit

The number of goblet cells in the duodenum and proximal ileum showed a significant difference (p ≤ 0.05) in treatments supplemented with GF compared to the CON. The GF4 group had the highest number of goblet cells compared to the other treatment groups, with the most significant value difference from the CON group. In the duodenum and proximal ileum, all the treatment groups had goblet cells with significantly larger sizes (p ≤ 0.05). Furthermore, the GF4 had the most extensive significant goblet cells (p ≤ 0.05) when compared to the other treatment groups.

### Viscera organ index

Table-3 shows that there was no difference in the numbers of the liver index in all treatment and CON groups. The heart index in GF2, GF3, and GF4 treatments was significantly higher (p ≤ 0.05), whereas the GF4 group produced the highest value and most significant compared to the CON (p ≤ 0.05). The spleen index has a significant difference (p ≤ 0.05) in all treatment groups compared to the CON, especially in the GF1 and GF3 which had the highest value. Intestinal weight index in all treatments showed no significant difference. Intestinal length has a significant difference (p ≤ 0.05) in all treatment groups when compared to the CON, especially in the GF4 group, with the highest value.

**Table-3:** Viscera organ index of broiler chickens fed with GF (Hornstedtia scottiana) treatments for 21 days.

Variables	Treatments					p-value

	Control	GF1	GF2	GF3	GF4	
Liver index	2.457 ± 0.378^ns^	2.321 ± 0.627^ns^	2.397 ± 0.482^ns^	2.229 ± 0.405^ns^	2.426 ± 0.926^ns^	0.539
Heart index	0.67 ± 0.011^a^	0.66 ± 0.035^a^	0.81 ± 0.026^bc^	0.75 ± 0.018^b^	0.85 ± 0.023^c^	0.001
Spleen index	0.061 ± 0.001^a^	0.083 ± 0.012^b^	0.086 ± 0.003^b^	0.088 ± 004^b^	0.094 ± 0.006^ab^	0.000
Intestine weight index	6.446 ± 4.158^ns^	6.626 ± 0.798^ns^	5.803 ± 0.526^ns^	5.951 ± 0.235^ns^	5.083 ± 0.085^ns^	0.361
Intestine length index	19.27 ± 0.681^ns^	20.47 ± 1.544^ns^	17.94 ± 0.565^ns^	19.29 ± 0.361^ns^	19.69 ± 1.485^ns^	0.569

Control: basal feed, GF1: 0.625%/kg basal feed, GF2: 1.25%/kg basal feed, GF3: 2.5%/kg basal feed, GF4: 5%/kg basal feed. ^ns^Means not significant. ^a-e^Different letters on values in the same row indicate a significant difference (p ≤ 0.05). *GF=Goloba kusi fruit

### Pectoralis major and gastrocnemius muscles

The effect of giving GF with various concentrations on the growth of the pectoralis muscle of broiler chickens is presented in Table-4. It was discovered that the weight of the pectoralis muscle, muscle index, fasciculus area, as well as the number and area of myofiber showed significant differences (p ≤ 0.05) in all treatment groups when compared to the CON. The GF4 had the highest value and was the most significantly different (p ≤ 0.05) group.

**Table-4:** Pectoralis major and gastrocnemius muscle morphology of broiler chickens fed with GF (*Hornstedtia scottiana*) treatments for 21 days.

Variables	Treatments					p-value

	Control	GF1	GF2	GF3	GF4	
Pectoralis major						
Muscle weight (g)	40.36 ± 1.02^a^	53.75 ± 2.61^b^	53.05 ± 1.62^b^	53.70 ± 0.22^b^	56.23 ± 2.24^b^	0.000
Muscle weight index	5.64 ± 0.33^a^	8.20 ± 0,52^b^	8.12 ± 0.36^b^	8.39 ± 0.21^b^	8.97 ± 0.26^b^	0.001
Muscle area (cm)	28.00 ± 1.52^a^	34.33 ± 2.33^b^	40.66 ± 1.76^c^	43.00 ± 1.52^c^	43.00 ± 1.52^c^	0.000
Fascicle area (μm^2^)	114151.33 ± 2574.76^a^	234432.16 ± 1946.13^c^	212639.83 ± 1845.02^b^	235681.21 ± 1293.10^c^	447776.07 ± 730.41^d^	0.002
Myofiber area (μm^2^)	561.00 ± 3.92^a^	841.96 ± 3.08^c^	666.43 ± 1.76^b^	843.54 ± 3.23^c^	853.53 ± 2.58^d^	0.000
Total myofiber	134.33 ± 1.45^a^	144.36 ± 1.20^b^	178.00 ± 1.028^c^	218.33 ± 1.20^d^	226.67 ± 1.85^e^	0.000
Gastrocnemius						
Muscle weight (g)	2.27 ± 0.13^a^	3.43 ± 0.13^b^	3.17 ± 0.30^b^	3.19 ± 0.27^b^	3.02 ± 0.14^b^	0.000
Muscle weight index	0.406 ± 0.02^a^	0.480 ± 0.02^b^	0.479 ± 0.00^b^	0.490 ± 0.03^b^	0.478 ± 0.01^b^	0.001
Fascicle area (μm^2^)	62024.02 ± 271.83^a^	133763.64 ± 368.38^b^	183540.81 ± 204.77^c^	203121.58 ± 355.49^d^	332745.24 ± 317.15^e^	0.000
Myofiber area (μm^2^)	630.06 ± 2.06^a^	753.00 ± 2.08^b^	766.38 ± 1.23^c^	829.65 ± 2.95^d^	861.00 ± 2.51^e^	0.000
Total myofiber	73.00 ± 1.15^a^	92.33 ± 1.20^b^	107.33 ± 1.20^c^	113.00 ± 1.52^d^	133.67 ± 1.20^e^	0.000

Control: basal feed, GF1: 0.625%/kg basal feed, GF2: 0.625%/kg basal feed, GF3: 0.625%/kg basal feed, GF4: 0.625%/kg basal feed. ^a-d^Different letters on values in the same row indicate a significant difference (p ≤ 0.05). *GF=Goloba kusi fruit

**Figure-2:**
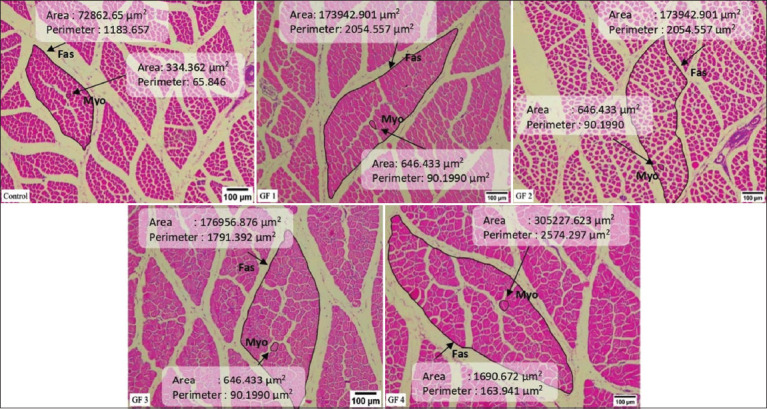
Transversal section fascicle (Fas) and myofiber (Myo) of pectoralis major muscle morphology of 21 days old broiler chicken treated with Goloba kusi fruit (GF) using 10 × 10 magnification. Control (CON): basal feed; GF1: GF1 0.625%/kg basal feed; GF2: GF2 1.25%/kg basal feed; GF3: GF3 2.5%/kg basal feed; GF4: GF4 5%/kg basal feed. This figure shows that the GF group had large size of fascicle compared to CON groups.

Table-4 shows the effect of giving GF on the growth of the gastrocnemius muscle of broilers. It was discovered that the weight of the gastrocnemius muscle, fascicle area, as well as the number and area of myofiber, have significantly different values (p ≤ 0.05) for all treatment groups. The GF4 group in the fasciculus area as well as the number and area of myofibers had the highest values than other groups. However, the gastrocnemius muscle index did not show a significant increase in the treatment group but the GF3 had the highest value. The morphology of the pectoralis major muscle in broiler chickens given GF supplements is illustrated in Figure-2.

### Broiler chicken growth performance

The effect of giving GF on the growth performance of broiler chickens is presented in Table-5. The increase in body weight in all treatment groups on day one did not have a significant difference. At the age of 6 days, all treatment groups had a significant increase in body weight (p ≤ 0.05) compared to the CON. The weight gain of the GF treatment group continued until 21 days and showed an increase (p ≤ 0.05) in all treatments, especially in the GF4 group, which has the highest value.

**Table-5:** Growth performance of broiler chickens fed with GF (*Hornstedtia scottiana*) treatments for 21 days.

Variables	Day	Treatments					p-value

		Control	GF1	GF2	GF3	GF4	
Body weight (g)	1	46.80 ± 1.17^ns^	47.00 ± 0.57^ns^	47.00 ± 0.57^ns^	47.00 ± 0.57^ns^	46.00 ± 0.57^ns^	0.684
	6	73.00 ± 0.60^a^	77.67 ± 1.52^b^	77.67 ± 1.52^b^	78.33 ± 1.52^b^	77.67 ± 1.52^b^	0.000
	10	165.00 ± 2.64^a^	175.00 ± 4.58^b^	178.00 ± 4.35^b^	176.33 ± 1.52^b^	179.00 ± 1.00^b^	0.000
	14	243.67 ± 1.52^a^	280.33 ± 2.08^b^	290.33 ± 1.52^c^	337.67 ± 7.37^d^	286.67 ± 1.52^bc^	0.001
	18	404.67 ± 2.51^a^	451.67 ± 1.15^b^	478.33 ± 1.52^c^	478.33 ± 1.52^c^	490.00 ± 8.00^d^	0.000
	21	542.67 ± 6.71^a^	626.67 ± 16.95^bc^	594.00 ± 28.05^b^	643.33 ± 12.00^bc^	665.67 ± 2.02^c^	0.002
Feed intake (g/day)		56.48 ± 0.36^c^	54.33 ± 2.18^b^	50.19 ± 2.18^a^	50.43 ± 1.80^a^	50.92 ± 1.04^a^	0.000
Weight gain (g/day)		24.31 ± 0.36^a^	27.46 ± 1.00^b^	28.24 ± 0.25^b^	27.89 ± 0.68^b^	29.02 ± 0.79^b^	0.000
Feed conversion ratio (g/day)		2.43 ± 0.09^b^	1.68 ± 0.34^ab^	1.69 ± 0.34^ab^	1.62 ± 0.28^ab^	1.47 ± 0.13^a^	0.043

Control: basal feed, GF1: 0.625%/kg basal feed, GF2: 0.625%/kg basal feed, GF3: 0.625%/kg basal feed, GF4: 0.625%/kg basal feed. ^ns^Means not significant. ^a-d^Different letters on values in the same row indicate a significant difference (p ≤ 0.05). *GF=Goloba kusi fruit

Feed intake of broiler chickens showed no difference in the CON and treatment groups. However, the CON group has the highest daily feed intake value compared to the GF treatments. The weight gain of broiler chickens treated with GF experienced a significant increase (p ≤ 0.05) for the GF1, GF2, GF3, and GF4 with values of 27.46 ± 1.00 g/day, 28.24 ± 0.25 g/day, 27.89 ± 0.68 g/day, and 29.02 ± 0.79 g/day, while the CON group has 24.31 ± 0.36 g/day. Daily weight gain in broilers fed with GF and CON does not correlate with daily feed consumption. This can be observed in Table-5, which shows the FCR value in the CON group, namely, 2.43 ± 0.09 g/day. The treatment group with the lowest FCR value of 1.47 ± 0.13 g/day was the GF4 group.

### Body morphometry and performance of broilers

The morphometric results of broiler chickens aged 1, 14, and 21 days are shown in Table-6. The morphometry of all characters measured at the age of 1 day did not have a significantly different value between each treatment group, compared to the CON. On the 14^th^ day, the height and breast circumference of chickens in all treatment groups had a significant difference (p ≤ 0.05) from the CON. Back length in the GF3 and GF4 groups had the most substantial value (p ≤ 0.05). Meanwhile, the other morphometric characters did not show a significant difference between the CON and treatment groups.

**Table-6:** Body morphometric measurements of broiler chickens fed with Goloba kusi fruit (*Hornstedtia scottiana*) treatments for 21 days.

Day	Variables (cm)	Treatments					p-value

		Control	GF1	GF2	GF3	GF4	
1	Body Length	11.31 ± 0.22^ns^	11.50 ± 0.09^ns^	10.65 ± 0.23^ns^	11.11 ± 0.29^ns^	11.05 ± 0.33^ns^	0.247
	Body height	10.69 ± 0.43^ns^	10.00 ± 0.50^ns^	10.70 ± 1.04^ns^	10.00 ± 1.00^ns^	10.43 ± 0.90^ns^	0.741
	Back length	6.83 ± 0.44^ns^	7.22 ± 0.36^ns^	7.13 ± 0.10^ns^	6.60 ± 0.05^ns^	6.63 ± 0.08^ns^	0.640
	Breast width	4.39 ± 0.20^ns^	4.38 ± 0.07^ns^	4.36 ± 0.07^ns^	4.20 ± 0.11^ns^	4.33 ± 0.12^ns^	0.814
	Chicken height	10.69 ± 0.43^ns^	10.00 ± 0.28^ns^	10.70 ± 0.60^ns^	10.00 ± 0.57^ns^	10.43 ± 0.52^ns^	0.740
	Thigh length	1.76 ± 0.14^ns^	1.80 ± 0.30^ns^	1.91 ± 0.05^ns^	2.04 ± 0.07^ns^	1.88 ± 0.09^ns^	0.768

**Day**	**Variables (cm)**	**Treatments**					**p-value**

		**Control**	**GF1**	**GF2**	**GF3**	**GF4**	

4	Body length	19.01 ± 0.13^ns^	19.75 ± 0.69^ns^	19.72 ± 0.14^ns^	19.77 ± 0.39^ns^	19.50 ± 0.28^ns^	0.240
	Body height	12.86 ± 0.31^a^	16.33 ± 0.76^b^	16.43 ± 0.74^b^	16.83 ± 0.44^b^	17.78 ± 0.51^b^	0.000
	Back length	10.82 ± 0.19^a^	10.77 ± 0.31^a^	10.53 ± 0.17^a^	12.00 ± 0.46^b^	12.06 ± 0.52^b^	0.030
	Breast width	11.80 ± 0.41^a^	13.05 ± 0.33^b^	13.26 ± 0.37^b^	13.46 ± 0.29^b^	14.06 ± 0.29^b^	0.001
	Chicken height	21.73 ± 0.63^ns^	22.33 ± 0.44^ns^	22.30 ± 0.19^ns^	21.40 ± 0.95^ns^	22.33 ± 0.45^ns^	0.771
	Thigh length	4.75 ± 0.21^ns^	5.22 ± 0.04^ns^	5.06 ± 0.76^ns^	5.13 ± 0.76^ns^	5.06 ± 0.76^ns^	0.230

**Day**	**Variables (cm)**	**Treatments**					**p-value**

		**Control**	**GF1**	**GF2**	**GF3**	**GF4**	

21	Body length	22.08 ± 0.22^a^	24.71 ± 0.28^c^	24.46 ± 0.24^bc^	23.26 ± 1.75^ab^	24.37 ± 0.23^bc^	0.005
	Body height	16.86 ± 0.44^a^	18.98 ± 0.65^a^	19.46 ± 0.35^a^	20.46 ± 0.86^a^	24.42 ± 2.10^b^	0.040
	Back length	12.73 ± 0.37^a^	14.26 ± 0.33^b^	14.80 ± 0.72^ab^	15.00 ± 0.87^ab^	15.60 ± 0.52^c^	0.004
	Breast width	13.25 ± 0.52^a^	15.26 ± 0.41^b^	17.40 ± 0.34^c^	18.00 ± 0.37^c^	18.26 ± 0.26^c^	0.000
	Chicken height	25.86 ± 0.46^a^	26.67 ± 0.33^ab^	27.50 ± 0.28^bc^	28.40 ± 0.45^cd^	29.03 ± 0.23^e^	0.000
	Thigh length	6.43 ± 0.29^a^	7.56 ± 0.28^b^	7.96 ± 0.43^b^	8.00 ± 0.23^b^	8.46 ± 0.29^b^	0.010

Control: basal feed, GF1: 0.625%/kg basal feed, GF2: 0.625%/kg basal feed, GF3: 0.625%/kg basal feed, GF4: 0.625%/kg basal feed. ^ns^Means not significant. ^a-c^Different letters on values in the same row indicate a significant difference (p≤0.05). *GF=Goloba kusi fruit

On day 21, chicken body length had the highest value in the GF1, GF2, and GF4 treatment groups, with a significant difference (p ≤ 0.05) from the CON group. The height of chickens for GF4 treatment had the highest value and the most important difference (p ≤ 0.05). Back length, chest circumference, and chicken height in all treatment groups also had significantly different values (p ≤ 0.05) from the CON group. Meanwhile, the thigh length was insignificant between the CON and the treatment groups.

## Discussion

Goloba kusi is the fruit of an endemic plant native to Indonesia and has long been used by the community as a food [5–7]; therefore, it is safe for consumption by humans and animals. The fruit has good nutrition when used as a feed substitute for broiler chickens without adverse effects on the body.

The small intestine is one part of the digestive system that functions to absorb nutrients [27]. The efficiency of absorption of dietary nutrients is influenced by the tissue structure in the small intestine, especially the length of the villi, the depth of the crypts, and the optimal number of goblet cells, to secrete mucin [28]. A healthy small intestine is also significant for optimal feed efficiency and better growth performance [29]. A well-functioning and healthy gut is essential for poultry [30]. When the function and health of the intestines are disturbed, digestion and absorption of nutrients are not optimal, thereby affecting the health and performance of poultry [31].

In this study, it was discovered that the length of the villi, the depth of the crypt duodenum and ileum, as well as the number and area of goblet cells, were better after the administration of GF. Table-2 shows that the GF4 group given 5% Goloba kusi experienced an increase compared to others. Villi in the small intestine improve the ability to absorb nutrients by increasing the intestine’s surface area [32]. The depth of the crypts plays a role in accelerating the repair of villous tissue damaged by sloughing, inflammation, or toxins from pathogens, indicating the broader area for the absorption of nutrients throughout the body [33]. The villi height is directly proportional to the crypt depth and can indicate small intestine morphology. Furthermore, a high villi/low crypt depth ratio shows good nutrient absorption [34]. Goblet cells also have an essential role in synthesizing fluid mucin, a glycoprotein that helps maintain intestinal ecology by expelling bacteria and preventing epithelial degradation [35]. The wider the goblet cell area and the greater the number of goblet cells, the more mucin is secreted, which assists nutrient absorption and protects the villous surface area from pathogen attack [28]. According to Uni *et al*. [36] and Deplance and Gaskins [37], goblet cells produce and secrete mucin which plays an essential role in brush border protection in nutrient absorption. Kavoi *et al*. [25] also reported that goblet cells secrete mucin in the digestive tract to protect the intestinal membrane from damage caused by enzymes and pathogens. Setiawan *et al*. [26] stated that the large area of villi and several goblet cells can enhance the nutrient absorption ability of livestock.

According to Myers *et al*. [38], flavonoids can increase the modulation of intestinal absorption and protect the digestive tract from damage caused by stress or pathogen attack. The Goloba kusi flavonoid test (*Horstedtia scottiana*) showed the presence of secondary metabolites in the form of flavonoids of 0.80% w/w, as presented in Table-7. Based on the results illustrated in Table-8, the nutritional content test contained 12.15% protein and 14.96% crude fiber. According to Prihambodo *et al*. [39], administering high doses of flavonoids can improve villi and increase villi height. Albab *et al*. [30] discovered that 10% (100 mg/mL) of flavonoids in Dates Water Extract (DWE) can increase the length of villi in the duodenum and jejunum. It can also increase the number and area of goblet cells in the duodenum, jejunum, and ileum, thereby affecting the absorption in the intestine. Ramadhanti *et al*. [40] stated that flavonoids contained in *Spirogyra jaoensis* extract can also increase villi height and goblet cell area in the duodenum jejunum and ileum, as well as the depth of the crypts in the jejunum and ileum to enhance nutrients absorption. Flavonoids can also stimulate the mitosis of epithelial cells in villi [41]. The previous studies have reported that the protein content in chicken feed significantly increases the digestive system’s efficiency [42, 43]. Boontiam *et al*. [44] reported that the administration of 0.05% lysophospholipid (LPL) containing crude protein, low energy, and amino acids in broiler DOC improves intestinal health and high villi in the jejunum. The administration of complex proteins can increase intestinal growth [45].

**Table-7:** Flavonoid and antioxidant test result of *Horstedtia scottiana*.

Variable	Result	Unit	Method
Total flavonoid	0,80	%b/b	UV-vis spectrophotometer

**Table-8:** Nutritional content of *Horstedtia scottiana*.

Parameter	Value
Crude protein (%)	12.15
Crude fiber (%)	14.96
Crude fat (%)	1.96
Ash (%)	7.00
Moisture (%)	12.15
Metabolizable energy (kcal/kg)	3642,490

The crude fiber content of 14.96% in GF also affects intestinal morphology and increases its effectiveness in nutrient absorption. This is in line with previous studies, where the presence of high crude fiber content of Trichoderma fermented wheat bran in feed improved the morphology of the small intestine in broiler chickens [46]. According to Saragih *et al*. [28], 37% of crude fiber contained in *S. jaoensis* positively impacts intestinal morphology and is effective in nutrient absorption.

Based on the study of Prihambodo *et al*. [39], the mechanism of flavonoids and proteins in increasing the length of the villi and the depth of the crypt is through the action of flavonoids as an antibacterial. It is carried out by inhibiting pathogenic bacteria in the small intestine that can injure intestinal villi and interfere with nutrient absorption [39]. A lower community of pathogenic bacteria can stimulate growth, regenerate intestinal villi, and intensify nutrient absorption [47]. According toRamadhanti *et al*. [40], flavonoids can also protect the small intestine wall of laying hens. Damiano *et al*. [48], the mechanism of action of flavonoids (quercetin) on goblet cells by stimulating goblet cells to increase the secretion of mucin, which is a glycoprotein, is essential to help the absorption of feed substances. The amount of mucin fluid in the goblet cells can be estimated from the cell’s area. Mucin fluid secreted by goblet cells will facilitate the absorption of nutrients in the small intestine [49]. Meanwhile, increased mucin in the small intestine and higher epithelial turnover can increase the effectiveness of the small intestine in nutrient absorption [50]. Optimal nutrient absorption will increase nutrient digestibility, including protein digestibility that can affect the duodenum related to protein function [44]. Brock *et al*. [51] also reported that proteins play a role in cell formation, replacing dead cells, signal transduction, cell growth and maintenance, and forming body tissues. Cells and body tissues formed include small intestinal epithelial cells [51, 52]. This indicated that the more epithelial cells in the small intestine, the wider the surface area and the greater the number of villi, increasing the ability to absorb nutrients. Proteins also play a role in controlling intestinal permeability, thereby facilitating the passage of ions as well as solutes intracellularly and playing a role in blocking the entry of unwanted antigens, toxins, and microorganisms [53].

The pectoralis and gastrocnemius muscles are striated muscles that tend to have fatness [54] and are an essential indicator of the successful growth of broiler chickens [28, 54]. From Table-4, providing adequate and appropriate GF containing crude protein and flavonoids can improve chicken muscle performance. Several studies report that protein is essential for muscle growth in chickens. Saragih and Daryono [55] stated that the provision of crude protein by 25% for 14 days can improve the performance of the pectoralis major muscle in DOC pelung and broiler chickens. According to Saragih *et al*. [28], the 16% protein content in *S*. *jaoensis* can increase the growth of the pectoralis muscle in broiler chickens. Liu *et al*. [56] discovered that 180 g/kg and 160 g/kg of crude protein given for 42 days increased chest muscle weight in Lueyang black bone chickens aged 42 days. Several studies have also reported that flavonoids can help improve muscle performance in chickens. Zhou *et al*. [57] revealed that flavonoids (baicalein) in the right dose can increase muscle growth in broilers aged 21–42 days. Based on the study by Albab *et al*. [30], the flavonoids contained in 10% (100 mg/mL) DWE given to layer chickens for 54 days yielded positive results on the growth of the pectoralis muscle, as shown from muscle weight, fascicle area, and myofiber area.

The mechanism of protein is helping muscle growth by stimulating the proliferation of satellite cells to regenerate myofiber for better muscle growth has also been reported [58]. According to Kim *et al*. [59], Branched-Chain Amino Acids are one of the primary regulators of protein synthesis. Their optimal ratio is essential for inducing nutrient sensors to signal myocyte proliferation and differentiation, leading to muscle growth, and development. Oskoueian *et al*. [60] stated that flavonoids (quercetin) at a dose of 200 mg/kg body weight can reduce oleic (18:1n-9), palmitate (16:0, 26.1%–27.9%), linoleic (18:2n-6), and stearic acid (18:0) in the pectoralis major muscle of broiler chickens. There is no information on the modulation of flavonoids (quercetin) by the fatty acid composition in meat. However, the secondary metabolites have an essential role as additional feed for broiler chickens to improve growth performance, especially in the pectoralis muscle [61].

Broiler chickens have fast body growth, which is influenced by several factors, such as feed efficiency [62] and nutrient content [42]. According to He *et al*. [12], protein plays an essential role in poultry growth, development, as well as health and is the main driver of broiler chickens’ feed intake and growth performance [52]. Moreover, flavonoid content also plays a role in growth in broiler chickens [58].

Tables-3 and 5 show that GF containing sufficient protein and the right flavonoids increased the growth performance indicators, namely, body weight, viscera organ index, and FCR values more efficiently than the CON. According to Srilatha *et al*. [63], 21% and 19% of crude protein can increase broiler chickens’ growth. Saragih *et al*. [28] also stated that 16% protein content in *S. jaoensis* improves body weight. Meanwhile, Boontiam *et al*. [44] discovered that administering 0.05% LPL containing crude protein in broiler DOC can improve growth performance. Perdamaian *et al*. [64] reported that 22% crude protein and energy metabolism of 3100 kcal/kg given for 7 days can increase the growth of DOC hybrid chickens. Saragih and Daryono [55] revealed that giving 25% crude protein for 14 days increases the body weight of broilers and pelung chickens. A previous study by Awad *et al*. [65] recorded that a 2% low crude protein diet added with glycine reduced body weight in broiler DOC, while Singh *et al*. [66] established that flavonoids can improve growth performance. Based on the results of Ouyang *et al*. [67], basal feed +15 mg/kg flavonoids contained in alfalfa (*Medicago sativa*) can significantly increase broiler body weight at 42 days compared to the basal diet. Zhou *et al*. [57] stated that flavonoids (baicalein) at a dose of 100–200 mg/kg increased the growth body weight of broiler chickens aged 21–42 days. Albab *et al*. [30] discovered that flavonoids in 10% (100 mg/mL) DWE given for 54 days increased body weight in layer chickens. According to Setiawan *et al*. [26], cashew leaf extract containing flavonoids given to DOC Jawa super chicken for 16 days improved body weight. The administration of kudzu leaf extract containing flavonoids to 1-day-old chicken also enhanced antioxidant capacity due to high Superoxide Dismutase activity and low malondialdehyde content. This can improve feed efficiency and benefit digestive health by increasing the diversity of gut bacteria and probiotic bacteria to enhance growth performance in chickens [68]. El-Far *et al*. [69] stated that flavonoids in 2% and 4% date palm seeds added to layer chicken feed can increase body weight and feed efficiency. Furthermore, Zhou *et al*. [57] discovered that flavonoids (baicalein) at a dose of 100–200 mg/kg increased the spleen index in broiler chickens aged 21–42 days. The investigation by Chen *et al*. [70] established that alfalfa (*M. sativa*) extract containing flavonoids can improve the spleen index in 21-day-old geese.

The FCR calculation in the GF4 treatment showed a significant efficiency compared to other treatment groups. The decreasing FCR value indicates that the rate of increase in growth is fast. According to Zhou *et al*. [57], flavonoids (baicalein) at 100–200 mg/kg can reduce FCR values in broilers aged 21–42 days. Albab *et al*. [30] also discovered that flavonoids in 10% (100 mg/mL) DWE given for 54 days significantly reduced the value of FCR in layer chicken DOC. Furthermore, Awad *et al*. [65] stated that the addition of a low-crude protein diet of 2% with glycine can increase the FCR value. Boontiam *et al*. [44] reported that the administration of 0.15% LPL containing crude protein, low energy, and amino acids for 5 weeks reduced the FCR value in broiler DOC.

Kamboh and Zhu [71] established that the mechanism of flavonoids and proteins improves growth performance by increasing the regulation of the combination of growth hormones and receptors in the liver. This leads to high concentrations of growth factors, such as insulin, that promote growth [71]. Beski *et al*. [42] stated that crude protein is an essential intake for the growth of broiler chickens and also improves insulin-like growth factor-1 messenger ribonucleic acid levels to increase growth in hybrid chickens [64]. Flavonoid and phenolic components play a role in inhibiting antibacterial activity and bacterial growth through several mechanisms, namely, interfering with bacterial respiration by absorbing metal ions and increasing the concentration of hydrogen peroxide, which causes oxidative stress [30]. A previous report showed that flavonoid acts as antibacterial through their oxidation potential and can donate hydrogen ions to neutralize the toxic effects of free radicals [72]. Furthermore, it increases the growth of chickens through the thalamus-pituitary axis, prevents the formation of free radicals, and improves the body’s resistance to stress [70].

Body morphometry is an important indicator of bone and muscle development [30]. In this study, the administration of GF for 21 days increased broiler body morphometry, including height, back length, chest width, and height. This indicates that GF positively affects broiler morphometry, as presented in Table-6. The results also showed that GF contains flavonoids and proteins, which enhanced the body morphometry of broiler chickens. Albab *et al*. [30] stated that the flavonoid content in 10% (100 mg/mL) DWE given for 54 days can improve the body morphometry of layer chicken DOC. According to Jubril *et al*. [73], administering 22% crude protein to quail for 5 weeks obtained better body morphometric results than other crude protein doses. Li *et al*. [74] reported that the development of internal organs affects the digestion and absorption of nutrients due to the effectiveness of nutrients in muscle and bone tissue.

Theprevious investigation stated that the mechanism of flavonoids and proteins in increasing broiler body morphometry begins with bone anabolic reactions through osteoblast differentiation on target molecules, namely p38 Mitogen-activated protein kinases [30]. The phosphorylation of p38 activates the Wnt signaling pathway, while the Wnt protein will bind to Frizzled’s Lipoprotein Receptor-associated Protein 5/6, signaling the cytosolic kinase glycogen synthase kinase-3 to be phosphorylated [30]. The Wnt protein can also prevent catenin-phosphorylation [75]. Furthermore, phosphorylated catenin that has been stabilized will be translocated to the nucleus and cooperate with transcription as well as lymphoid enhancing or T cell factors to increase transcription of genes involved in bone differentiation of the Runx2 gene [75].

## Conclusion

The results showed that the addition of 5% GF in basal feed significantly improves the small intestine morphology, muscle morphology, and broiler chicken growth performance. This indicates that GF can be used as a natural feed additive, containing sufficient crude protein, crude fiber, and secondary metabolites such as flavonoids to improve the growth performance of broiler chickens. The limitation of this study is that only the tissue morphology and performance were measured, while the expression of gene level was not explored. Therefore, it is recommended that future investigations should focus more on the expression of gene level.

## Authors’ Contributions

HTS and DB: Designed and contributed to the experimentation. HTS, DB, and NS: Conducted the experiment, drafted, and edited the article. All authors have read, revised, and approved the final manuscript.
